# Construction and validation of a risk prediction model for early ventilator-induced diaphragm dysfunction in mechanically ventilated patients

**DOI:** 10.3389/fmed.2026.1861008

**Published:** 2026-06-25

**Authors:** Min-Qing Long, Yu-Ting Liu

**Affiliations:** Department of Critical Care Medicine, Huazhong University of Science and Technology Union Shenzhen Hospital/Shenzhen Nanshan People’s Hospital, Shenzhen, Guangdong, China

**Keywords:** mechanical ventilation, nomogram, temporal validation, ultra-early risk prediction, ventilator-induced diaphragm dysfunction

## Abstract

**Background:**

Mechanical ventilation is a core life-support technique in intensive care units (ICUs), but it can easily induce ventilator-induced diaphragm dysfunction (VIDD) in the early stage of mechanical ventilation (within 24 h). VIDD is defined as new-onset diaphragm dysfunction caused by mechanical ventilation, and diaphragm ultrasound is the gold standard for its diagnosis. This study aimed to explore the risk factors of early VIDD in mechanically ventilated patients and construct a temporally validated risk prediction model using clinical baseline indicators.

**Methods:**

A prospective study was conducted on 470 mechanically ventilated patients who were admitted to the ICU of Shenzhen Nanshan People’s Hospital from May 2024 to May 2025. Patients were divided into a modeling group (*n =* 328) and a temporal validation group (*n =* 142) at a 7:3 ratio. VIDD was diagnosed by the simultaneous fulfillment of three ultrasound indicators (AND logic): diaphragm thickening fraction (TFdi) < 20%, diaphragm excursion(DE) < 1 cm, and diaphragm thickness(Tdi) < 0.2 cm. These ultrasound indicators served exclusively as the outcome diagnostic criteria, not as predictive variables. Based on the incidence of VIDD, patients were categorized into VIDD and non-VIDD groups. In total, 34 potential predictive variables were collected, including general data, mechanical ventilation-related parameters, medication use, treatment methods, laboratory test indicators, and relevant rating scales. Independent risk factors were identified via Lasso regression directly without univariate pre-screening, followed by multivariate logistic regression. R software was applied to construct a nomogram model. The discriminative ability, calibration, and clinical validity of the prediction model were evaluated using the area under the receiver operating characteristic (ROC) curve (AUC), calibration curve, and decision curve analysis (DCA).

**Results:**

Among 328 patients in the modeling group, 160 developed VIDD, with an incidence rate of 48.78%. Independent risk factors for VIDD included complicated sepsis, controlled mechanical ventilation mode, Richmond agitation-sedation scale (RASS) score ≤ − 4, acute physiology and chronic health evaluation II (APACHE II) score >20, and complicated intra-abdominal hypertension (IAP ≥ 16 mmHg) (all *p* < 0.05). No diaphragm ultrasound-derived indicators were included in the final model, as they were used for outcome diagnosis rather than predictive variables. The nomogram model constructed based on the above factors indicated an AUC of 0.820 (95% CI: 0.775 ~ 0.865) in the modeling group, with a Youden index of 0.514, a sensitivity of 0.806 (0.745, 0.867), and a specificity of 0.708 (0.640, 0.777). The AUC of the temporal validation group was 0.816 (95% CI: 0.748 ~ 0.885), with a Youden index of 0.564, a sensitivity of 0.783 (0.685, 0.880), and a specificity of 0.781 (0.686, 0.876), suggesting excellent discriminative ability and favorable external stability of the model. In the Hosmer-Lemeshow test (modeling group: *χ*^2^ = 4.957, *p* = 0.549; validation group: *χ*^2^ = 8.431, *p* = 0.208), the calibration curve indicated a high degree of agreement between the predicted values and the actual values. The DCA threshold probabilities of the model were 0.11–0.88 and 0.13–0.77 in the modeling and validation groups, respectively, suggesting a wide range of beneficial thresholds and good clinical validity.

**Conclusion:**

The nomogram model constructed based on the five risk factors exhibited favorable efficacy for predicting early VIDD. Therefore, the model can be applied as a convenient quantitative tool for the early identification of high-risk patients in clinical practice.

## 1 Introduction

Mechanical ventilation represents a crucial supportive treatment to save the lives of critically ill patients in the intensive care unit (ICU). Globally, nearly half of ICU-admitted patients require mechanical ventilation support, and the utilization rate of mechanical ventilation in ICU settings reaches 73% in China (1, 2). While stabilizing the vital signs of patients, mechanical ventilation may induce a serious yet often overlooked complication, namely ventilator-induced diaphragm dysfunction (VIDD) Diaphragm dysfunction in critically ill patients includes three distinct but overlapping entities: critical illness-related diaphragm dysfunction (CIDD), sepsis-associated diaphragm dysfunction (SIDD), and ventilator-induced diaphragm dysfunction (VIDD). CIDD is caused by multiple pathways of critical illness; SIDD is driven by sepsis-induced inflammatory injury; VIDD is specifically defined as new-onset diaphragm dysfunction directly caused by mechanical ventilation, independent of baseline critical illness or sepsis (3). Using ultrasound imaging, studies have shown that 53% of critically ill patients develop diaphragm dysfunction within 24 h of initiating mechanical ventilation, and this proportion may rise to 79% within 72 h of mechanical ventilation (4). Several studies have reported that diaphragm dysfunction increases the risk of extubation failure by 8 times, prolongs ICU stay by 40%, increases the risk of mortality by 2 times, and elevates the direct medical cost per patient by approximately 25,000 US dollars, imposing a heavy burden on patient prognosis and medical resource allocation (5–7).

Given the rapid progression and high risk of VIDD within the initial 24 h of mechanical ventilation, it is crucial to advance monitoring and intervention. Considering the advantages of non-invasiveness, high sensitivity, and dynamic assessment, diaphragm ultrasound has become an ideal tool for the early diagnosis of diaphragm dysfunction in mechanically ventilated (MV) patients (8–11). However, most previous prediction models for diaphragm dysfunction were limited to single-disease cohorts, focused on 48–72 h after ventilation, and lacked standardized ultrasound diagnostic criteria. In addition, no prior model has focused on the 0–24 h ultra-early window of mechanical ventilation. Furthermore, existing models often rely on ultrasound indicators as predictors, which limits their utility in settings where immediate ultrasound expertise is unavailable. Therefore, this study aimed to employ standardized diaphragm ultrasound as the gold standard for outcome diagnosis, and to screen readily available clinical baseline risk factors to construct a temporally validated nomogram for the ultra-early prediction of VIDD. This approach is intended to provide a practical bedside screening tool that enables risk stratification before ultrasound assessment, thus facilitating the earliest possible initiation of diaphragm-protective interventions.

## 2 Materials and methods

### 2.1 Study design and population

A prospective observational study design was adopted, and 470 mechanically ventilated patients who were admitted to the ICU ward of a Grade A tertiary hospital in Shenzhen from May 2024 to May 2025 were enrolled as research subjects. The inclusion criteria were as follows: ① age ≥18 years old; ② expected duration of mechanical ventilation ≥24 h. The exclusion criteria were as follows: ① presence of diaphragm dysfunction before mechanical ventilation; ② pregnancy; ③ contraindications to diaphragm ultrasound or inability to cooperate with the examinations, such as severe thoracoabdominal deformity, large-area thoracoabdominal dressing coverage, and unclear display of diaphragm structure on ultrasound images, etc.); ④ patients who withdrew from the study midway, transferred to another hospital, or died within 24 h; ⑤ complicated with severe underlying diseases, such as severe liver and kidney failure, coagulation disorders, etc., which could affect diaphragm function assessment or prognosis; ⑥ refusal to sign the informed consent form.

According to the Hanley formula for sample size calculation (12) and referring to a recent prediction model for diaphragm dysfunction among critically ill patients (13), with an expected AUC = 0.80, *δ* = 0.04, and Z = 1.96 (*α* = 0.05), the sample size was determined to be 384 cases. Considering a 20% loss to follow-up rate, the expected sample size was increased to 460.8, and we finally included 470 cases. Furthermore,

the final model included 5 predictors with 160 VIDD events, resulting in 32 events per predictor(EPP), which fully meets the ≥10 EPP rule recommended for prediction model development to ensure statistical stability. According to the 7:3 principle, 328 cases were included in the modeling group and 142 cases were assigned to the temporal validation group. Grouping was determined based on the admission time sequence of the research subjects. Data from 328 mechanically ventilated patients from May 2024 to February 2025 were used as the modeling group for the construction and temporal validation of the nomogram model. From March 2025 to May 2025, 142 cases were assigned to the temporal validation group for external validation of the model. Temporal validation refers to model validation in a subsequent cohort from the same center, which is a recognized and recommended strategy for prediction model validation. External validation in an independent cohort from a different center remains a necessary step for future generalization. This study protocol was reviewed and approved by the Ethics Committee of Shenzhen Nanshan People’s Hospital (approval number: KY-2024-102201).

### 2.2 Measurement methods and diagnostic criteria for diaphragm dysfunction

Before starting the study, all personnel conducting diaphragm ultrasound held certificates of intensive care ultrasound technical assessment and completed unified operational standard training. Intra-observer reliability was assessed by repeated measurements in 30 patients (ICC = 0.89), and inter-observer reliability was evaluated by two independent investigators (ICC = 0.86), indicating good measurement consistency to ensure the accuracy of VIDD diagnosis. After starting the study, medical staff systematically assessed the enrolled mechanically ventilated patients. Both the modeling and validation groups completed diaphragm ultrasound examinations and collected various clinical indicators within 24 h of mechanical ventilation. Diaphragm ultrasound was conducted using a portable color Doppler ultrasound system ME7 Exp (Mindray, Shenzhen). After turning on the device and entering patient information, a 6 ~ 13 MHz linear probe was used, and the M mode was selected for evaluation. A superficial probe or vascular probe was adopted. When conditions permitted, the patient was placed in a 30° head-up supine position, and the probe was placed vertically to the chest wall at the attachment point of the right diaphragm and the chest wall (surface localization near the 8th ~ 10 intercostal space of the right midaxillary line) to detect diaphragm contraction and relaxation. The diaphragm was located deep in the chest wall soft tissue and on the surface of the liver, with a hypoechoic area between two approximately parallel hyperechoic lines (located in the parietal pleura and peritoneum, respectively). The distance between the parietal pleura and the peritoneum was regarded as the diaphragm thickness (Tdi). The normal reference value of Tdi for adults is 0.22 ~ 0.28 cm. End-expiratory and end-inspiratory time points were confirmed based on the ventilator flow curve. End-expiratory Tdi and end-inspiratory Tdi were measured after freezing the image. Each operator measured 2 breathing cycles and took the average value. The diaphragm thickening fraction (TFdi) was calculated using the following formula: Diaphragm thickening fractio*n =* (end-inspiratory diaphragm thickness - end-expiratory diaphragm thickness)/end-expiratory diaphragm thickness × 100%. Meanwhile, diaphragm movement was measured to obtain the vertical distance between end-expiratory and end-inspiratory diaphragm, which is known as diaphragm excursion (DE). Consistent with Chinese and international intensive care ultrasound guidelines and consensus (14, 15), to reduce the false-positive rate and ensure diagnostic accuracy, VIDD was diagnosed only when all three ultrasound indicators were abnormal simultaneously (AND logic): TFdi <20%, AND DE < 1 cm, AND Tdi < 0.2 cm. Thereafter, all patients were divided into the non-VIDD and VIDD groups. Relevant indicators were collected in the validation group, and VIDD diagnosis was made within 24 h of mechanical ventilation. Predictive evaluation was conducted using the constructed risk prediction model. Validation of model performance was conducted by comparing the predicted results with the actual diagnosis results.

### 2.3 Study instruments

Thirty-four candidate predictors were selected based on the pathophysiology of early VIDD, published evidence, and bedside clinical availability, following literature review and expert consultation. These factors were specifically divided into 6 categories: ① general characteristics including age, gender, body mass index (BMI), nutritional method, complicated intra-abdominal hypertension (IAP ≥ 16 mmHg), extracted from the medical records of patients upon ICU admission; ② Mechanical ventilation-related parameters, including positive end-expiratory pressure (PEEP), mechanical ventilation mode, oxygenation index, partial pressure of carbon dioxide in arterial blood (Paco2), which were extracted from real-time monitoring records and blood gas analysis results during the first episode of mechanical ventilation. Mechanical ventilation modes were clearly defined before analysis to avoid classification bias: Control mode included Volume Control Ventilation (VCV) and Pressure Control Ventilation (PCV); Assist mode included Pressure Support Ventilation (PSV) and Synchronized Intermittent Mandatory Ventilation (SIMV) + PSV. All patients received standardized ventilation settings (PEEP 5–15 cmH₂O, tidal volume 6–8 mL/kg) in the first 24 h based on our institutional protocol. ③ Clinical management factors: whether sepsis was diagnosed; use of sedative drugs, their duration of use, and relevant Richmond agitation-sedation scale (RASS) score; use of analgesic drugs, their duration of use, and critical care pain observation tool (CPOT); use of glucocorticoids and their duration of use; restricted activity treatments, such as continuous renal replacement therapy (CRRT), extracorporeal membrane oxygenation (ECMO), prone position ventilation, intra-aortic balloon pump (IABP), extracted from the first diagnosis and treatment records within 24 h of ICU admission and medications, operations, and scoring records during treatment; ④ laboratory markers. Including C-reactive protein (CRP), lactic acid (Lac), serum sodium (Na+), serum calcium (Ca2+), serum potassium (K+), Serum phosphate (Pi), serum albumin (Alb), blood glucose, and hemoglobin (HGB). All indicators were obtained from early morning fasting venous blood tests collected when patients were admitted to the ICU; ⑤ severity scales: Glasgow Coma scale (GCS score) within 24 h of admission, acute physiology and chronic health evaluation II (APACHE II score) within 24 h of admission, sequential organ failure assessment (SOFA score) within 24 h of admission, activities of daily living (ADL score) within 24 h of admission, nutritional risk screening (NRS score) within 24 h of admission, extracted from the medical records of patients upon ICU admission; ⑥ other data: being complicated with diseases involving neuromuscular lesions, undergoing surgery on organs adjacent to the diaphragm (cardiac, thoracic, abdominal surgery), extracted from the medical records of patients upon ICU admission. A broad inclusion, strict selection strategy was used to avoid pre-selection bias, fully cover VIDD-related pathogenic pathways, and ensure all variables are rapidly obtainable at the bedside for clinical application.

### 2.4 Quality control

① Control of selection bias: We strictly implement the established inclusion and exclusion criteria to screen research subjects and prevent the inclusion of non-target populations. All researchers participated in unified training covering research objectives, indicator collection standards, data recording requirements, etc. They could only participate in the research implementation after passing theoretical and practical assessments. ② Control of information bias: A double-entry mode was adopted for data management, with two independent researchers entering clinical data. Cross-validation of data consistency was conducted after data entry, and entries with discrepancies were traced back to original records for verification and correction to ensure the accuracy and completeness of data entry.

### 2.5 Statistical analysis

All analyses were performed using SPSS 23.0 and R 4.0.2. Normality was assessed using the Shapiro–Wilk test; skewed variables were presented as median (interquartile range, IQR) and compared using Mann–Whitney U test, while normally distributed variables were presented as mean ± standard deviation (SD) and compared using independent t-test. Categorical variables were compared using *χ*^2^ test. To avoid excluding clinically important predictors that exert effects only in multivariable combinations, candidate variables were selected based on clinical relevance and prior evidence, and screened directly by Lasso regression without univariate pre-screening. Optimal *λ* value was determined by 10-fold cross-validation. Variables with non-zero coefficients in the Lasso model were then entered into multivariate logistic regression analysis to identify independent risk factors. Multicollinearity was assessed using variance inflation factor (VIF), with VIF < 5 indicating no significant collinearity. Model performance was evaluated by the area under the ROC curve (AUC), calibration curve with the Hosmer-Lemeshow test, and decision curve analysis (DCA). A *p*-value < 0.05 was considered statistically significant.

## 3 Results

### 3.1 General data of the study population

**Figure 1:**
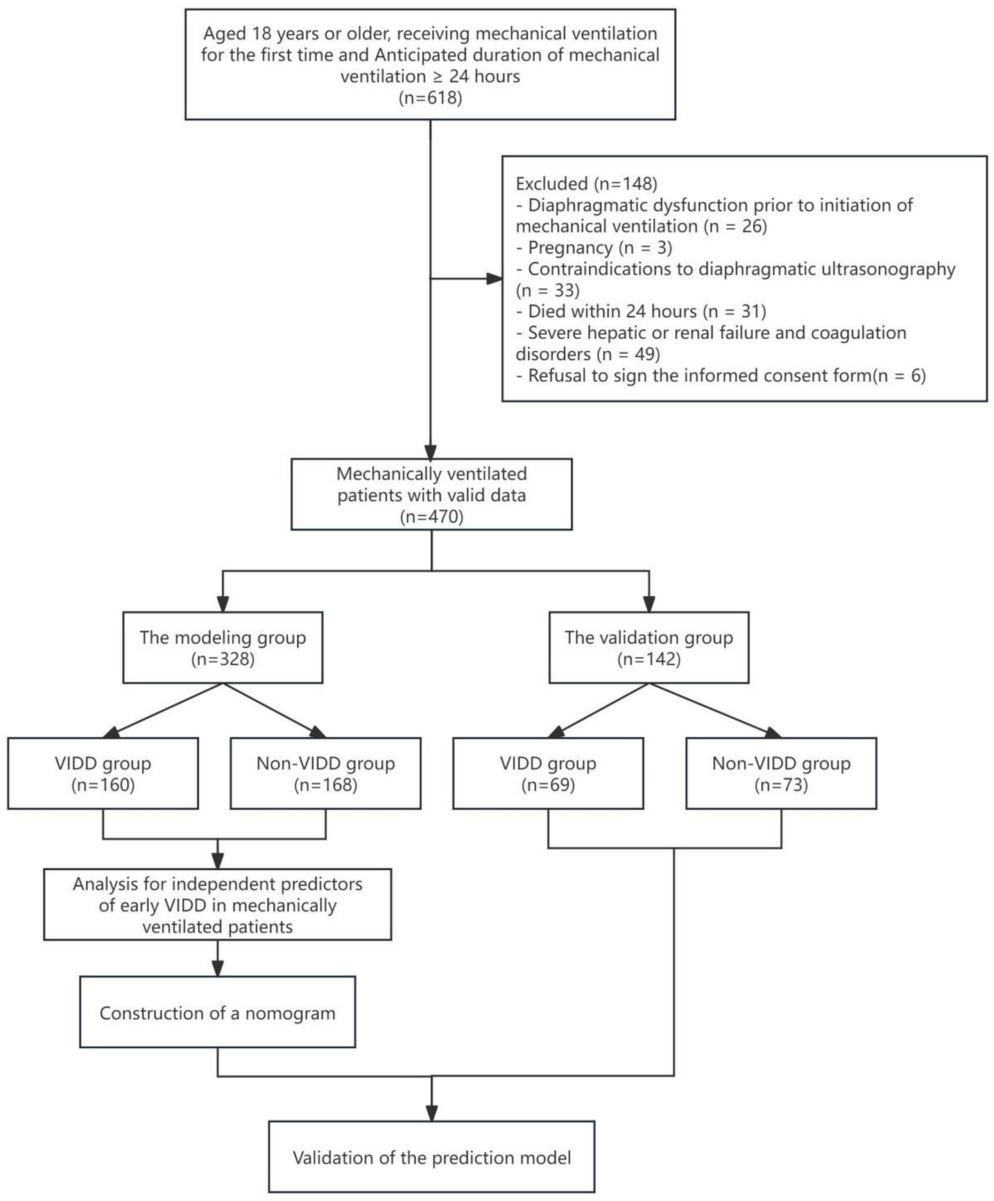
Patient enrollment flowchart. Patients aged ≥18 years, receiving mechanical ventilation with expected duration ≥24 h were initially assessed (*n =* 618). Excluded (*n =* 148): pre-existing diaphragm dysfunction (*n =* 26); pregnancy (*n =* 3); contraindications to diaphragm ultrasound (*n =* 33); death within 24 h (*n =* 31); severe hepatic/renal failure or coagulation disorders (*n =* 49); refusal to consent (*n =* 6). Finally, 470 eligible patients were enrolled: 328 in the modeling cohort and 142 in the temporal validation cohort.

In total, 328 patients were included in the modeling group, including 222 males and 106 females (Figure 1). Among them, 160 developed VIDD, with an incidence rate of 48.78% (160/328). Furthermore, 142 patients were included in the temporal validation group, comprising 94 males and 48 females. Among them, 69 developed VIDD, with an incidence rate of 48.59% (69/142). There were no significant differences between the modeling and validation groups in terms of clinical indicators (*p* > 0.05), suggesting that the data were comparable (Table 1).

**Table 1:** Comparison of early VIDD in mechanically ventilated patients between the modeling and validation groups.

Variable	Modeling group (*n =* 328)	Validation group (*n =* 142)	Statistic	*P*-value
Age [years, *n* (%)]	–	–	0.376^*^	0.540
≤65.0	181 (55.2)	74 (52.1)	–	–
>65.0	147 (44.8)	68 (47.9)	–	–
Sex [*n* (%)]	–	–	0.099^*^	0.753
Male	222 (67.7)	94 (66.2)	–	–
Female	106 (32.3)	48 (33.8)	–	–
Body mass index (BMI) [kg/m^2^, mean ± SD]	23.88 ± 3.82	23.92 ± 3.83	−0.122^△^	0.903
APACHE II score [points, *n* (%)]	–	–	2.981^*^	0.084
≤20.0	129 (39.3)	68 (47.9)	–	–
>20.0	199 (60.7)	74 (52.1)	–	–
SOFA score [points, mean ± SD]	4.41 ± 4.05	4.15 ± 3.72	0.664^△^	0.507
NRS 2002 score [points, *n* (%)]	–	–	0.010^*^	0.919
≤3.0	255 (77.7)	111 (78.2)	–	–
>3.0	73 (22.3)	31 (21.8)	–	–
Activities of Daily Living (ADL) score [points, *n* (%)]	–	–	0.723^*^	0.696
≤40	226 (68.9)	95 (66.9)	–	–
41–60	70 (21.3)	35 (24.6)	–	–
61–100	32 (9.8)	12 (8.5)	–	–
Intra-abdominal pressure (IAP) [mmHg, *n* (%)]	–	–	1.596^*^	0.206
<16	107 (32.6)	38 (26.8)	–	–
≥16	221 (67.4)	104 (73.2)	–	–
Positive end-expiratory pressure (PEEP) [*n* (%)]	–	–	0.785^*^	0.376
≤15.0	266 (81.1)	120 (84.5)	–	–
>15.0	62 (18.9)	22 (15.5)	–	–
Oxygenation index [mmHg, mean ± SD]	281.63 ± 68.99	281.67 ± 67.92	−0.006^△^	0.995
Arterial partial pressure of carbon dioxide (PaCO₂) [mmHg, mean ± SD]	40.27 ± 6.05	39.64 ± 5.73	1.059^△^	0.290
Mechanical ventilation mode [*n* (%)]	–	–	3.713^*^	0.054
Assist mode	15 (4.6)	13 (9.2)	–	–
Control mode	313 (95.4)	129 (90.8)	–	–
Sepsis [*n* (%)]	–	–	0.161^*^	0.688
No	89 (27.1)	36 (25.4)	–	–
Yes	239 (72.9)	106 (74.6)	–	–
Nausea [*n* (%)]	–	–	0.822^*^	0.365
No	222 (67.7)	90 (63.4)	–	–
Yes	106 (32.3)	52 (36.6)	–	–
Vomiting [*n* (%)]	–	–	0.108^*^	0.742
No	191 (58.2)	85 (59.9)	–	–
Yes	137 (41.8)	57 (40.1)	–	–
Sedative use [*n* (%)]	–	–	0.431^*^	0.512
No	74 (22.6)	36 (25.4)	–	–
Yes	254 (77.4)	106 (74.6)	–	–
Analgesic use [*n* (%)]	–	–	1.477^*^	0.224
No	43 (13.1)	13 (9.2)	–	–
Yes	285 (86.9)	129 (90.8)	–	–
Glucocorticoid use [*n* (%)]	–	–	0.241^*^	0.623
No	179 (54.6)	74 (52.1)	–	–
Yes	149 (45.4)	68 (47.9)	–	–
Continuous renal replacement therapy (CRRT) [*n* (%)]	–	–	1.359^*^	0.244
No	250 (76.2)	101 (71.1)	–	–
Yes	78 (23.8)	41 (28.9)	–	–
Extracorporeal membrane oxygenation (ECMO) [*n* (%)]	–	–	1.100^*^	0.294
No	299 (91.2)	125 (88)	–	–
Yes	29 (8.8)	17 (12)	–	–
Prone position ventilation [*n* (%)]	–	–	0.059^*^	0.808
No	271 (82.6)	116 (81.7)	–	–
Yes	57 (17.4)	26 (18.3)	–	–
Intra-aortic balloon pump (IABP) [*n* (%)]	–	–	1.682^*^	0.195
No	275 (83.8)	112 (78.9)	–	–
Yes	53 (16.2)	30 (21.1)	–	–
Nutrition route [*n* (%)]	–	–	1.885^*^	0.390
Fasting	107 (32.6)	52 (36.6)	–	–
Enteral nutrition	148 (45.1)	66 (46.5)	–	–
Parenteral nutrition	73 (22.3)	24 (16.9)	–	–
Neuromuscular disorders [*n* (%)]	–	–	1.549^*^	0.213
No	266 (81.1)	108 (76.1)	–	–
Yes	62 (18.9)	34 (23.9)	–	–
Cardiac surgery [*n* (%)]	–	–	0.923^*^	0.337
No	265 (80.8)	120 (84.5)	–	–
Yes	63 (19.2)	22 (15.5)	–	–
Thoracic surgery [*n* (%)]	–	–	0.092^*^	0.761
No	274 (83.5)	117 (82.4)	–	–
Yes	54 (16.5)	25 (17.6)	–	–
Abdominal surgery [*n* (%)]	–	–	2.644^*^	0.104
No	203 (61.9)	99 (69.7)	–	–
Yes	125 (38.1)	43 (30.3)	–	–
Blood lactate [mmol/L, mean ± SD]	7.32 ± 2.12	7.34 ± 2.24	−0.068^△^	0.945
C-reactive protein (CRP) [mg/L, mean ± SD]	92.46 ± 68.67	91.41 ± 64.90	0.154^△^	0.878
Serum sodium [mmol/L, mean ± SD]	139.97 ± 6.24	139.49 ± 6.27	0.755^△^	0.451
Serum calcium [mmol/L, mean ± SD]	2.11 ± 0.43	2.06 ± 0.40	1.272^△^	0.204
Serum potassium [mmol/L, mean ± SD]	4.74 ± 0.97	4.63 ± 0.93	1.150^△^	0.251
Serum phosphate [mmol/L), mean ± SD]	1.18 ± 0.32	1.16 ± 0.30	0.611^△^	0.542
Serum albumin [g/L, mean ± SD]	30.32 ± 7.59	31.43 ± 7.90	−1.442^△^	0.150
Blood glucose [mmol/L, mean ± SD]	6.83 ± 2.82	6.95 ± 2.79	−0.394^△^	0.694
Hemoglobin (HGB) [g/L, mean ± SD]	90.50 ± 18.05	90.32 ± 18.25	0.099^△^	0.921
Sedative duration [h, *n* (%)]	–	–	2.012^*^	0.366
None	54 (16.5)	30 (21.1)	–	–
<12	114 (34.8)	42 (29.6)	–	–
≥12	160 (48.8)	70 (49.3)	–	–
Type of sedative [*n* (%)]	–	–	3.341^*^	0.342
None	74 (22.6)	36 (25.4)	–	–
Benzodiazepines	118 (36)	52 (36.6)	–	–
Non-benzodiazepines	86 (26.2)	27 (19)	–	–
Neuromuscular blocking agents	50 (15.2)	27 (19)	–	–
Richmond Agitation-Sedation Scale (RASS) score [*n* (%)]	–	–	1.718^*^	0.190
>−4	235 (71.6)	110 (77.5)	–	–
≤ − 4	93 (28.4)	32 (22.5)	–	–
Type of analgesic [*n* (%)]	–	–	2.208^*^	0.331
None	43 (13.1)	13 (9.2)	–	–
Opioid analgesics	133 (40.5)	66 (46.5)	–	–
Non-opioid analgesics	152 (46.3)	63 (44.4)	–	–
Analgesic duration [h, *n* (%)]	–	–	2.042^*^	0.360
None	43 (13.1)	13 (9.2)	–	–
<12	117 (35.7)	58 (40.8)	–	–
≥12	168 (51.2)	71 (50)	–	–
Critical-Care Pain Observation Tool (CPOT) score [*n* (%)]	–	–	3.321^*^	0.345
None	43 (13.1)	13 (9.2)	–	–
0–2	66 (20.1)	36 (25.4)	–	–
3–4	125 (38.1)	58 (40.8)	–	–
5–8	94 (28.7)	35 (24.6)	–	–
Glucocorticoid duration [h, *n* (%)]	–	–	5.671^*^	0.059
None	179 (54.6)	74 (52.1)	–	–
<12	50 (15.2)	34 (23.9)	–	–
≥12	99 (30.2)	34 (23.9)	–	–

^*^For the *χ*^2^ value; ^†^for the Mann–Whitney U test (skewed continuous variables: SOFA score, blood lactate);△for the t value; VIDD, ventilator-induced diaphragm dysfunction; BMI, body mass index; APACHE II, Acute Physiology and Chronic Health Evaluation II; SOFA, Sequential Organ Failure Assessment; NRS, Nutritional Risk Screening; ADL, Activities of Daily Living; IAP, intra-abdominal pressure; PEEP, positive end-expiratory pressure; PaCO₂, arterial partial pressure of carbon dioxide; CRRT, continuous renal replacement therapy; ECMO, extracorporeal membrane oxygenation; IABP, intra-aortic balloon pump; CRP, C-reactive protein; HGB, hemoglobin; RASS, Richmond Agitation-Sedation Scale; CPOT, Critical-Care Pain Observation Tool; SD, standard deviation; IQR, interquartile range.

### 3.2 Baseline characteristics of the modeling cohort

Baseline characteristics between the non-VIDD group (*n =* 168) and VIDD group (*n =* 160) are presented in Table 2. Skewed continuous variables (SOFA score, blood lactate) were described as median (interquartile range, IQR), and normally distributed continuous variables were described as mean ± standard deviation (SD). Univariate comparisons were conducted for descriptive purposes only and were not used for variable pre-screening in model development.

**Table 2:** Comparison of early VIDD in mechanically ventilated patients.

Variable	Non-VIDD group (*n =* 168)	VIDD group(*n =* 160)	Statistic	*P*-value
Age [years, *n* (%)]	–	–	3.393^*^	0.065
≤65.0	101 (60.12)	80 (50.00)	–	–
>65.0	67 (39.88)	80 (50.00)	–	–
Sex [*n* (%)]	–	–	0.605^*^	0.437
Male	117 (69.64)	105 (65.62)	–	–
Female	51 (30.36)	55 (34.38)	–	–
Body mass index (BMI) [kg/m^**2**^, mean ± SD]	23.56 ± 3.52	24.21 ± 4.11	−1.533^△^	0.126
APACHE II score [points, *n* (%)]	–	–	58.862^*^	<0.001
≤20.0	100 (59.52)	29 (18.12)	–	–
>20.0	68 (40.48)	131 (81.88)	–	–
SOFA score [points, mean ± SD]	3.99 ± 4.02	4.86 ± 4.05	−1.948^△^	0.052
NRS 2002 score [points, *n* (%)]	–	–	0.011^*^	0.917
≤3.0	131 (77.98)	124 (77.50)	–	–
>3.0	37 (22.02)	36 (22.50)	–	–
Activities of Daily Living (ADL) score [points, *n* (%)]	–	–	1.881^*^	0.390
≤40	114 (67.86)	112 (70.00)	–	–
41–60	34 (20.24)	36 (22.50)	–	–
61–100	20 (11.90)	12 (7.50)	–	–
Intra-abdominal pressure (IAP) [mmHg, *n* (%)]	–	–	16.414^*^	<0.001
≤16	72 (42.86)	35 (21.88)	–	–
>16	96 (57.14)	125 (78.12)	–	–
Positive end-expiratory pressure (PEEP) [*n* (%)]	–	–	6.103^*^	0.013
≤15.0	145 (86.31)	121 (75.62)	–	–
>15.0	23 (13.69)	39 (24.38)	–	–
Oxygenation index [mmHg, mean ± SD]	282.59 ± 64.78	280.62 ± 73.34	0.257^△^	0.797
Arterial partial pressure of carbon dioxide (PaCO₂) [mmHg, mean ± SD]	40.21 ± 6.70	40.34 ± 5.31	−0.203^△^	0.839
Mechanical ventilation mode [*n* (%)]	–	–	11.158^*^	<0.001
Assist mode	14 (8.33)	1 (0.62)	–	–
Control mode	154 (91.67)	159 (99.38)	–	–
Sepsis [*n* (%)]	–	–	20.928^*^	<0.001
No	64 (38.10)	25 (15.62)	–	–
Yes	104 (61.90)	135 (84.38)	–	–
Nausea [*n* (%)]	–	–	1.028^*^	0.311
No	118 (70.24)	104 (65.00)	–	–
Yes	50 (29.76)	56 (35.00)	–	–
Vomiting [*n* (%)]	–	–	0.236^*^	0.627
No	100 (59.52)	91 (56.88)	–	–
Yes	68 (40.48)	69 (43.12)	–	–
Sedative use [*n* (%)]	–	–	7.121^*^	0.008
No	48 (28.57)	26 (16.25)	–	–
Yes	120 (71.43)	134 (83.75)	–	–
Analgesic use [*n* (%)]	–	–	0.948^*^	0.330
No	25 (14.88)	18 (11.25)	–	–
Yes	143 (85.12)	142 (88.75)	–	–
Glucocorticoid use [*n* (%)]	–	–	3.405^*^	0.065
No	100 (59.52)	79 (49.38)	–	–
Yes	68 (40.48)	81 (50.62)	–	–
Continuous renal replacement therapy (CRRT) [*n* (%)]	–	–	0.586^*^	0.444
No	131 (77.98)	119 (74.38)	–	–
Yes	37 (22.02)	41 (25.62)	–	–
Extracorporeal membrane oxygenation (ECMO) [*n* (%)]	–	–	1.233^*^	0.267
No	156 (92.86)	143 (89.38)	–	–
Yes	12 (7.14)	17 (10.62)	–	–
Prone position ventilation [*n* (%)]	–	–	7.186^*^	0.007
No	148 (88.10)	123 (76.88)	–	–
Yes	20 (11.90)	37 (23.12)	–	–
Intra-aortic balloon pump (IABP) [*n* (%)]	–	–	0.066^*^	0.798
No	140 (83.33)	135 (84.38)	–	–
Yes	28 (16.67)	25 (15.62)	–	–
Nutrition route [*n* (%)]	–	–	0.046^*^	0.977
Fasting	54 (32.14)	53 (33.12)	–	–
Enteral nutrition	76 (45.24)	72 (45.00)	–	–
Parenteral nutrition	38 (22.62)	35 (21.88)	–	–
Neuromuscular disorders [*n* (%)]	–	–	0.605^*^	0.437
No	139 (82.74)	127 (79.38)	–	–
Yes	29 (17.26)	33 (20.62)	–	–
Cardiac surgery [*n* (%)]	–	–	1.433^*^	0.231
No	140 (83.33)	125 (78.12)	–	–
Yes	28 (16.67)	35 (21.88)	–	–
Thoracic surgery [*n* (%)]	–	–	0.244^*^	0.621
No	142 (84.52)	132 (82.50)	–	–
Yes	26 (15.48)	28 (17.50)	–	–
Abdominal surgery [*n* (%)]	–	–	0.212^*^	0.645
No	106 (63.10)	97 (60.62)	–	–
Yes	62 (36.90)	63 (39.38)	–	–
Blood lactate [mmol/L, mean ± SD]	7.49 ± 2.08	7.15 ± 2.14	1.438^△^	0.151
C-reactive protein (CRP) [mg/L, mean ± SD]	89.36 ± 65.34	95.71 ± 72.06	−0.837^△^	0.403
Serum sodium [mmol/L, mean ± SD]	140.20 ± 6.26	139.72 ± 6.23	0.701^△^	0.484
Serum calcium [mmol/L, mean ± SD]	2.16 ± 0.41	2.06 ± 0.44	2.313^△^	0.021
Serum potassium [mmol/L, mean ± SD]	4.77 ± 0.97	4.71 ± 0.97	0.551^△^	0.582
Serum phosphate [mmol/L, mean±SD]	1.15 ± 0.31	1.21 ± 0.33	1.642	0.101
Serum albumin [g/L, mean ± SD]	30.59 ± 7.73	30.03 ± 7.45	0.662^△^	0.508
Blood glucose [mmol/L, mean ± SD]	6.54 ± 2.81	7.14 ± 2.81	−1.938^△^	0.053
Hemoglobin (HGB) [g/L, mean ± SD]	91.40 ± 17.00	89.55 ± 19.11	0.924^△^	0.356
Sedative duration [h, *n* (%)]	–	–	4.101^*^	0.129
None	28 (16.67)	26 (16.25)	–	–
<12	50 (29.76)	64 (40.00)	–	–
≥12	90 (53.57)	70 (43.75)	–	–
Type of sedative [*n* (%)]	–	–	9.722^*^	0.021
None	48 (28.57)	26 (16.25)	–	–
Benzodiazepines	56 (33.33)	62 (38.75)	–	–
Non-benzodiazepines	45 (26.79)	41 (25.62)	–	–
Neuromuscular blocking agents	19 (11.31)	31 (19.38)	–	–
Richmond Agitation-Sedation Scale (RASS) score [*n* (%)]	–	–	18.679^*^	<0.001
>−4	138 (82.14)	97 (60.62)	–	–
≤ − 4	30 (17.86)	63 (39.38)	–	–
Type of analgesic [*n* (%)]	–	–	1.238^*^	0.538
None	25 (14.88)	18 (11.25)	–	–
Opioid analgesics	69 (41.07)	64 (40.00)	–	–
Non-opioid analgesics	74 (44.05)	78 (48.75)	–	–
Analgesic duration [h, *n* (%)]	–	–	2.019^*^	0.364
None	25 (14.88)	18 (11.25)	–	–
<12	63 (37.50)	54 (33.75)	–	–
≥12	80 (47.62)	88 (55.00)	–	–
Critical-Care Pain Observation Tool (CPOT) score [*n* (%)]	–	–	1.733^*^	0.630
None	25 (14.88)	18 (11.25)	–	–
0–2	30 (17.86)	36 (22.50)	–	–
3–4	64 (38.10)	61 (38.12)	–	–
5–8	49 (29.17)	45 (28.12)	–	–
Glucocorticoid duration [h, *n* (%)]	–	–	3.573^*^	0.168
None	100 (59.52)	79 (49.38)	–	–
<12	24 (14.29)	26 (16.25)	–	–
≥12	44 (26.19)	55 (34.38)	–	–

*For the *χ*^2^ value; ^†^for the Mann–Whitney U test (skewed continuous variables: SOFA score, blood lactate); △for the *t-*value; VIDD, ventilator-induced diaphragm dysfunction; BMI, body mass index; APACHE II, Acute Physiology and Chronic Health Evaluation II; SOFA, Sequential Organ Failure Assessment; NRS, Nutritional Risk Screening; ADL, Activities of Daily Living; IAP, intra-abdominal pressure; PEEP, positive end-expiratory pressure; PaCO₂, arterial partial pressure of carbon dioxide; CRRT, continuous renal replacement therapy; ECMO, extracorporeal membrane oxygenation; IABP, intra-aortic balloon pump; CRP, C-reactive protein; HGB, hemoglobin; RASS, Richmond Agitation-Sedation Scale; CPOT, Critical-Care Pain Observation Tool; SD, standard deviation; IQR, interquartile range.

### 3.3 Lasso regression analysis

**Figure 2:**
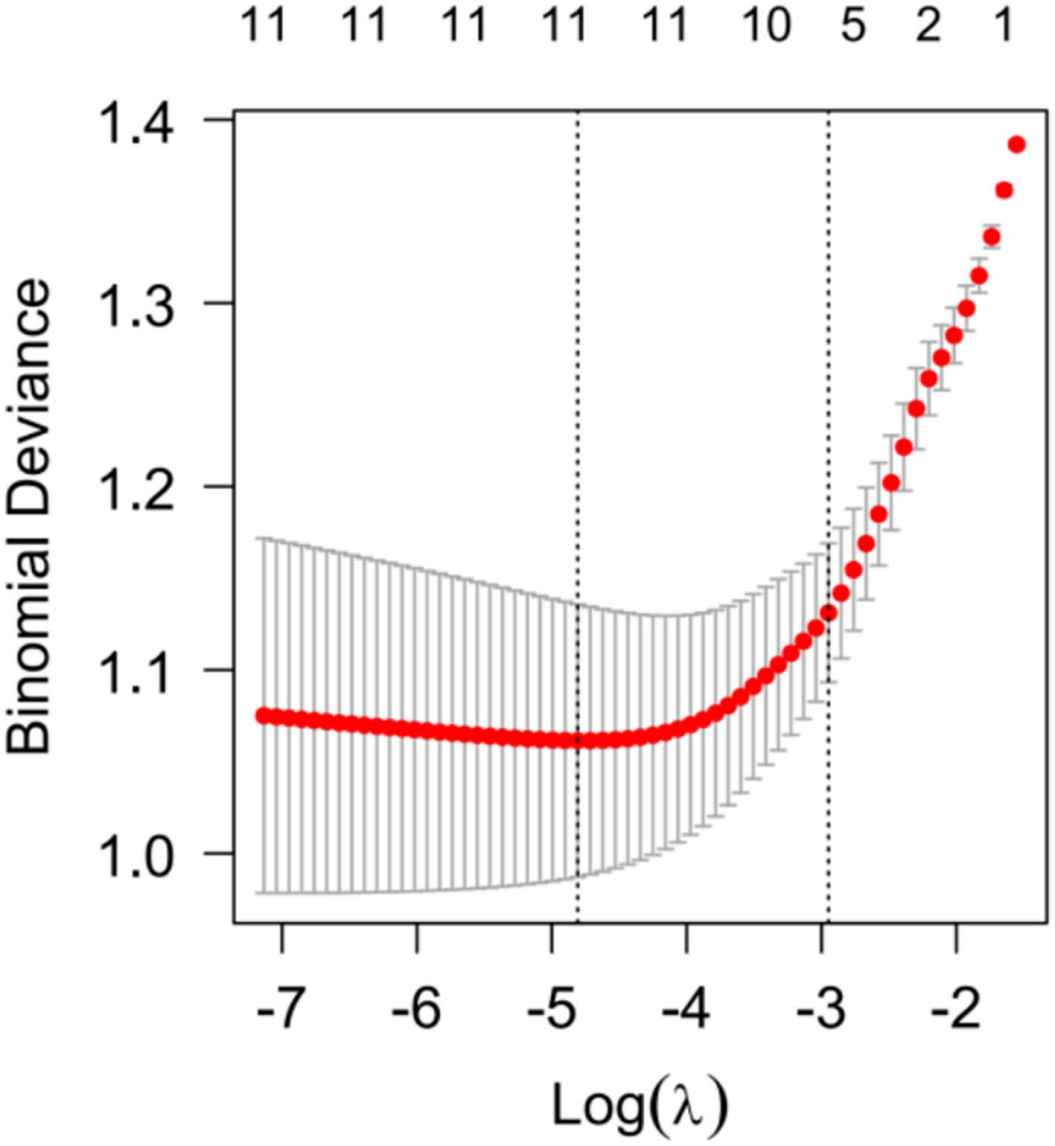
Selection of the optimal regularization parameter *λ* via 10-fold cross-validation in Lasso regression.

**Figure 3:**
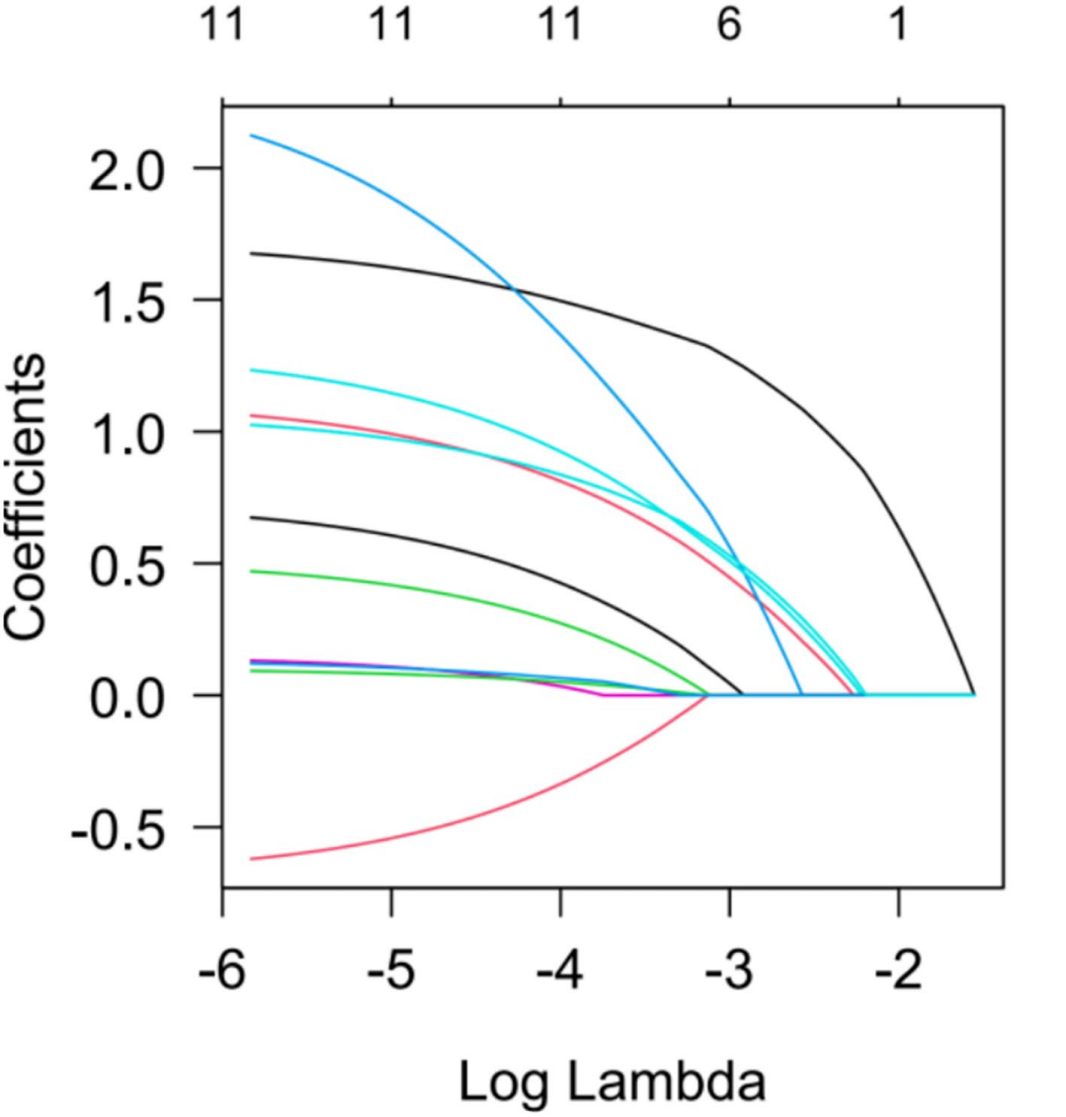
Variable selection path of Lasso regression for 6 candidate predictors.

All 34 candidate variables were included in Lasso regression analysis directly, without univariate pre-screening. Ten-fold cross-validation was adopted to determine the optimal regularization parameter *λ* = 0.05245. Six variables entered the initial candidate set, including APACHE II score (1.257), intra-abdominal hypertension ≥16 mmHg (0.419), mechanical ventilation mode (0.503), complicated sepsis (0.482), RASS score (0.503), and prone position ventilation (0.036) (Figures 2, 3). Among them, prone position ventilation had the smallest absolute Lasso coefficient (0.017) and was preferentially compressed to 0 with the increase of *λ* value, indicating that its predictive contribution to VIDD risk was extremely low; therefore, it was not included in subsequent multivariate Logistic regression analyses.

### 3.4 Multivariate analysis of early diaphragm dysfunction

Taking the incidence of VIDD within the first 24 h of mechanical ventilation as the dependent variable and the 5 variables selected using Lasso regression as independent variables, multivariate logistic regression analysis was performed. The relevant assignment information is presented in Table 3. All VIF values were < 5, indicating no substantial collinearity among the predictors. The regression analysis indicated that complicated sepsis, controlled mechanical ventilation mode, RASS score ≤ − 4, APACHE II score >20, and complicated intra-abdominal hypertension (IAP ≥ 16 mmHg) were independent risk factors for VIDD within the first 24 h of mechanical ventilation (all *p* < 0.05) (Table 4). No diaphragm ultrasound indicators entered the final model, as they were used to define the outcome (VIDD) rather than baseline predictive variables collected before VIDD occurrence.

**Table 3:** Variable coding for multivariate logistic regression analysis.

Variable	Coding
APACHE II score	≤20 = 0; >20 = 1
IAP	<16 mmHg = 0; ≥16 mmHg = 1
Mechanical ventilation mode	Assist mode = 0; Control mode = 1
Sepsis	No = 0; Yes = 1
RASS score	> − 4 = 0; ≤ − 4 = 1

APACHE II, Acute Physiology and Chronic Health Evaluation II; IAP, intra-abdominal pressure; RASS, Richmond Agitation-Sedation Scale.

**Table 4:** Risk factors for early VIDD in mechanically ventilated patients using multivariate logistic regression analysis.

Variable	*β*	SE	Wald *χ*^2^	*P*-value	OR (95% CI)
APACHE II score >20	1.840	0.290	6.346	<0.001	6.296 (3.567–11.114)
IAP ≥ 16 mmHg	1.098	0.293	3.753	<0.001	2.999 (1.690–5.322)
Controlled mechanical ventilation	2.438	1.079	2.260	0.024	11.450 (1.383–94.812)
Sepsis	1.181	0.319	3.705	<0.001	3.258 (1.744–6.084)
RASS score ≤ − 4	1.187	0.311	3.810	<0.001	3.276 (1.779–6.031)

### 3.5 Establishment of a nomogram prediction model for early diaphragm dysfunction in mechanically ventilated patients

**Figure 4:**
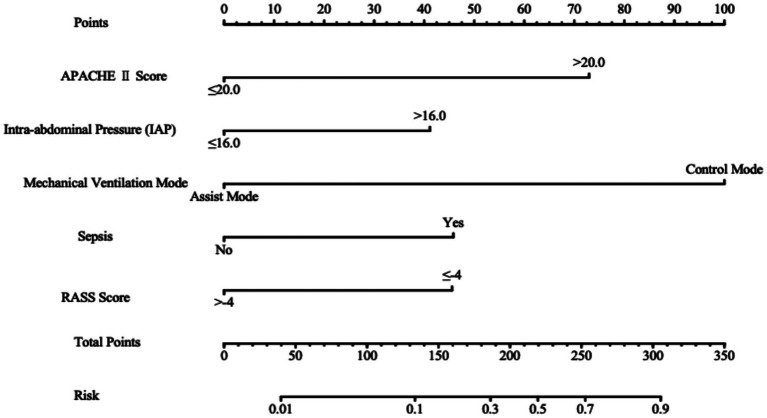
Nomogram for predicting the risk of early VIDD in mechanically ventilated patients.

Based on the 5 independent predictors selected using logistic regression analysis, a nomogram was constructed to predict the risk of VIDD within the first 24 h of mechanical ventilation (Figure 4). The total score was calculated by summing the scores corresponding to the predictors in the figure, and “VIDD occurrence risk” was the predicted probability. The higher the total score, the higher the risk of VIDD within the first 24 h of mechanical ventilation. The prediction model formula was Z = −5.612 + 1.840 (APACHE II score >20) + 1.098 (intra-abdominal pressure ≥16 mmHg) + 2.438 (controlled mechanical ventilation mode) + 1.181 (sepsis) + 1.187 (RASS score ≤ − 4).

### 3.6 Alidation of the nomogram model for early diaphragm dysfunction in mechanically ventilated patients

**Figure 5:**
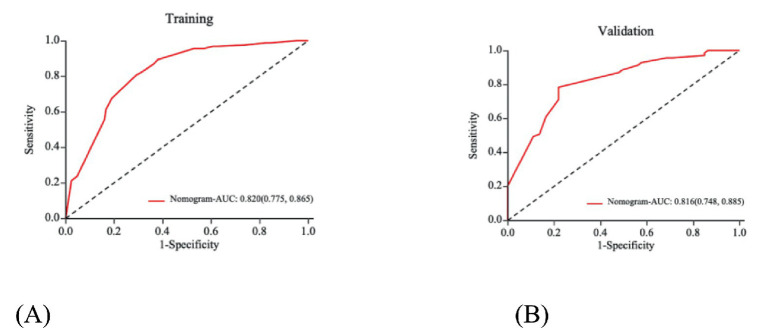
Receiver operating characteristic (ROC) curves of the prediction model. **(A)** Training cohort. **(B)** Temporal validation cohort.

**Figure 6:**
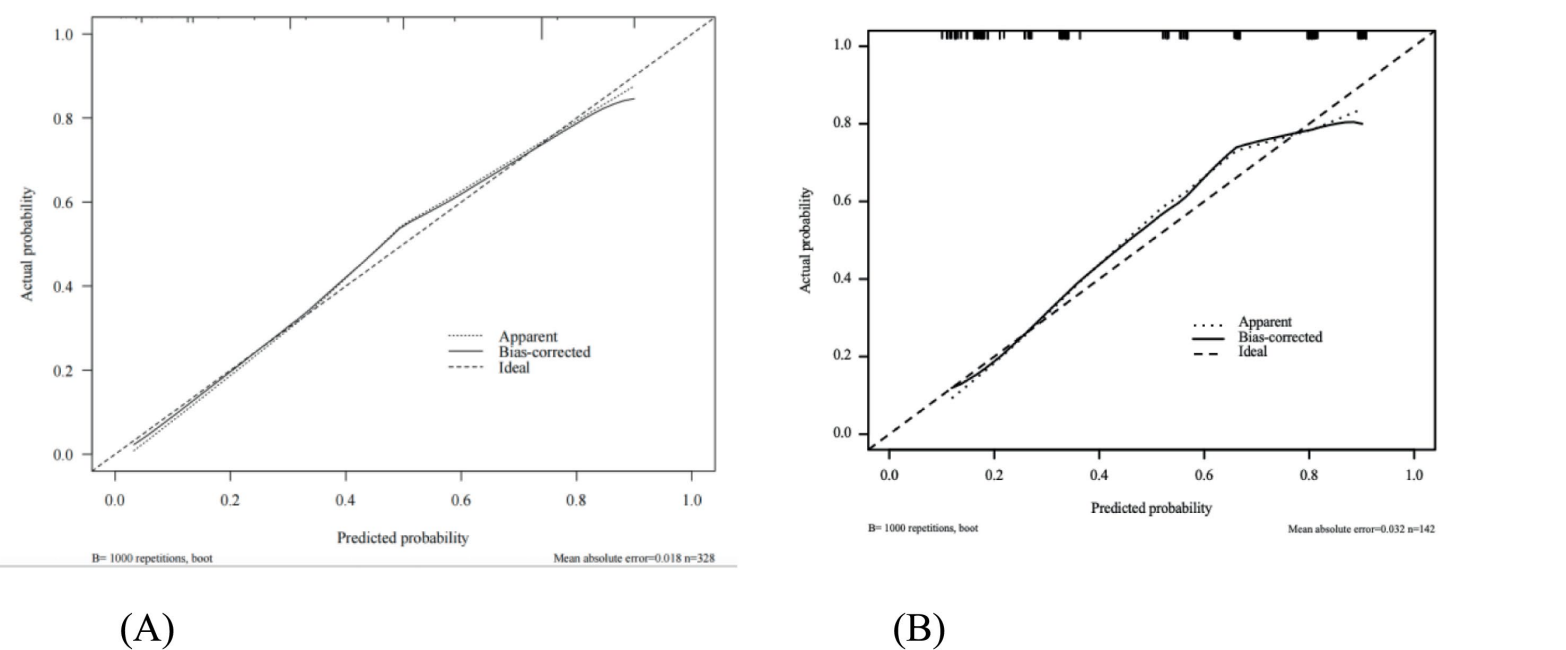
Calibration curves of the nomogram model. **(A)** Training cohort. **(B)** Temporal validation cohort.

**Figure 7:**
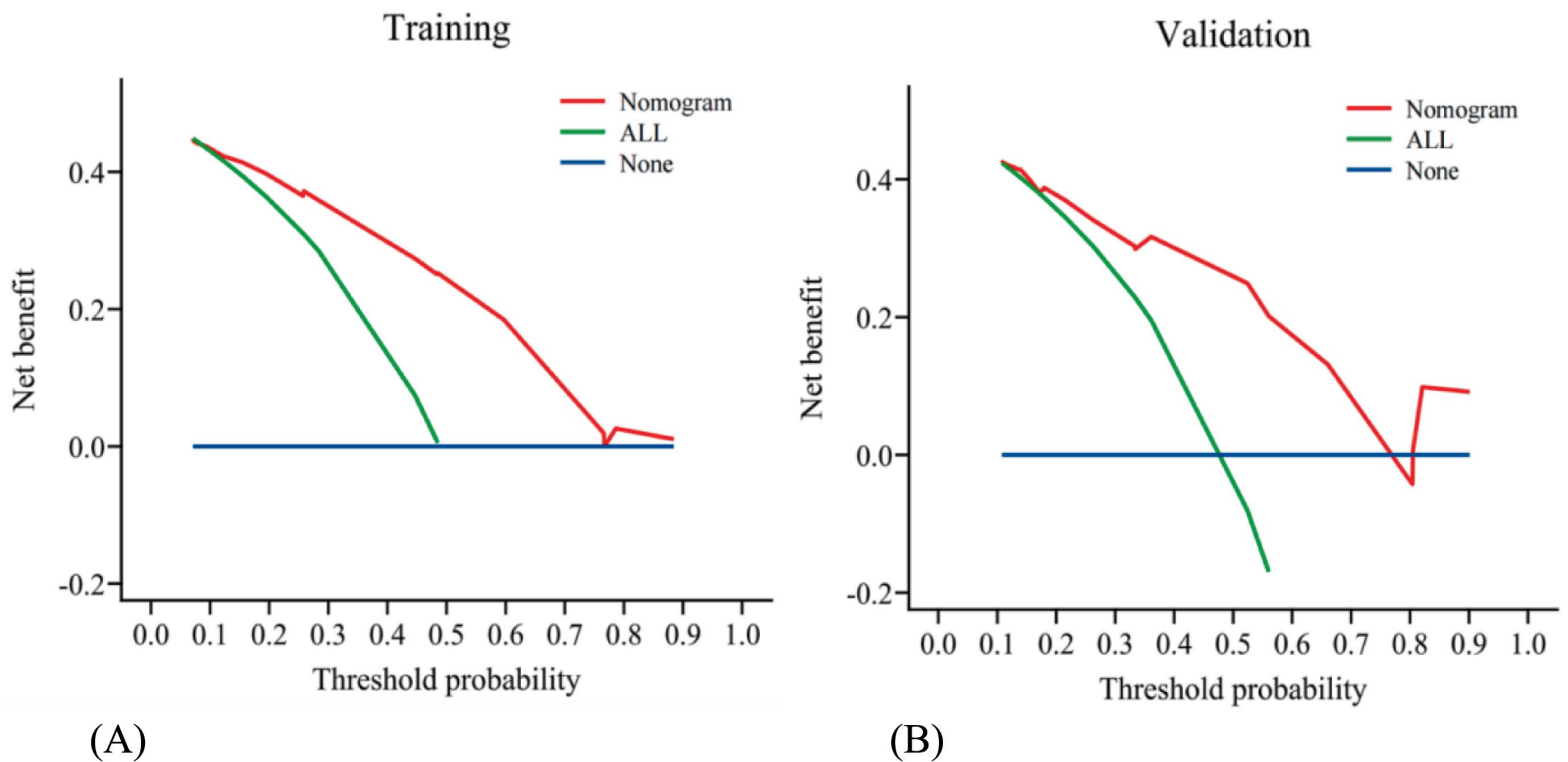
Decision curve analysis (DCA) of the prediction model. **(A)** Training cohort. **(B)** Temporal validation cohort.

The predictive performance of the prediction model was evaluated by drawing the ROC curve. The AUC of the ROC curve corresponding to the modeling group was 0.820 (95%CI: 0.775 ~ 0.865), its optimal cutoff value was 0.484, its Youden index was 0.514, its sensitivity was 0.806, and its specificity was 0.708. The AUC of the validation group was 0.816 (95%CI: 0.748 ~ 0.885), its optimal cutoff value was 0.348, its Youden index was 0.564, its sensitivity was 0.783, and its specificity was 0.781 (Figure 5). Temporal validation revealed that among 142 patients, 69 actually developed VIDD with an incidence rate of 48.59%, and the model predicted 54 cases of VIDD (sensitivity = 0.783). In addition, 73 patients did not actually develop VIDD, and the model predicted 57 cases (specificity = 0.781). Therefore, the overall prediction accuracy of the model was (54 + 57)÷142 ≈ 78.17%. Combined with the results of AUC, sensitivity, and specificity, the model exhibited good discriminative ability. The AUC values of both the modeling group and the validation group were >0.8, suggesting that the model offered good discriminative ability. The calibration degree of the model was evaluated based on the calibration curve and Hosmer-Lemeshow test. The results showed that: modeling group Hosmer-Lemeshow test *χ*^2^ = 4.957, *p* = 0.549; validation group *χ*^2^ = 8.431, *p* = 0.208, both *p* >0.05. Besides, the calibration curves of the modeling group and the validation group were highly consistent with the ideal curve (Figure 6). This finding suggests that the predicted values of the model were highly consistent with the actual occurrence values of VIDD, and the calibration ability was good. In addition, the clinical net benefit of the model was evaluated using DCA. The results showed that within the entire range of threshold probability 0.0 ~ 1.0, the DCA threshold probabilities of the model in the modeling and validation groups were 0.11–0.88 and 0.13–0.77, respectively (Figure 7). This finding indicates that the model had a wide range of beneficial thresholds and good clinical validity.

## 4 Discussion

### 4.1 High incidence of early VIDD in mechanically ventilated patients

This study indicated that the incidence of VIDD was 48.78% within the first 24 h of mechanical ventilation, which was similar to the results of Wang Xinchao et al. (16) (43.95%) and slightly lower than the result of Demoule et al. (4) (53%).

This difference may be attributed to the diagnostic criteria of VIDD in different studies, disease severity of the study population, the diagnosis and treatment level of different medical institutions, and initial intervention measures (17). Compared with previous models, our study has three incremental novelties: (1) targeting an unselected broad ICU cohort; (2) using strict AND-logic ultrasound diagnostic criteria; (3) constructing a purely clinical predictor model for wider bedside application.

Based on the traditional view, VIDD is a complication in the late stage of mechanical ventilation, but the latest pathophysiological studies have shown that diaphragmatic disuse atrophy and oxidative stress injury start within 12 ~ 24 h of initiating mechanical ventilation. The pathogenesis of VIDD depends on mechanical ventilation-mediated multi-link synergistic injury, with the mechanical ventilation mode serving as the initiating factor (18). Full-support controlled ventilation will lead to complete disuse of the diaphragm. Even partial-support assist ventilation will reduce the diaphragm load. Both will result in the rapid disuse atrophy of the diaphragm, and the cross-sectional area of diaphragmatic fibers may decrease by 15% ~ 20% within 12 ~ 18 h, and the atrophy further worsens within 24 h (19, 20). At the same time, oxidative stress induced by mechanical ventilation upregulates the production of mitochondrial reactive oxygen species and downregulates the antioxidant capacity of the body, thereby activating several proteolytic pathways, inhibiting protein synthesis, and leading to the metabolic imbalance of the diaphragm (21, 22). In addition, the diaphragm itself is specifically susceptible to disuse injury, and synergistic factors, such as infection and electrolyte disturbance, worsen disuse injury in critically ill patients, explaining the progression of VIDD from molecular injury to functional abnormality within 24 h. The results of this study suggest that clinical attention should be paid to early protection of the diaphragm during mechanical ventilation. Herein, the visualized nomogram model constructed in this study provides a convenient and quantitative tool for the rapid identification of high-risk VIDD populations in clinical practice.

### 4.2 The factors associated with early VIDD in mechanically ventilated patients

#### 4.2.1 Mechanically ventilated patients with APACHE II score >20 are prone to early VIDD

The APACHE II score is a widely used tool for evaluating disease severity in ICU-admitted patients. The higher the score, the more severe changes in physiological parameters, and the acute physiological function decline (23). This study revealed that the risk of VIDD in patients with APACHE II score >20 was 6.30 times that of patients with score ≤20 within 24 h of mechanical ventilation, which is consistent with the results of several studies (24, 25). A severe disease state corresponding to a high APACHE II score can promote the development of diaphragm dysfunction by inducing extensive physiological function disorders, metabolic imbalance, inflammatory response, etc. Previous studies have confirmed that patients with high APACHE II scores in ICU are more prone to the rapid loss of diaphragm strength and structural diaphragmatic damage. This damage will be further aggravated with mechanical ventilation, ultimately inducing early VIDD. Therefore, medical staff should pay attention to the APACHE II score of mechanically ventilated patients upon admission and correct the abnormal physiological indicators of patients in a timely manner, which is of great significance for reducing the risk of VIDD and improving patient prognosis.

#### 4.2.2 Mechanically ventilated patients with complicated intra-abdominal hypertension are prone to early VIDD

This study found that the risk of VIDD in mechanically ventilated patients with complicated intra-abdominal hypertension was significantly increased within 24 h of mechanical ventilation, which was consistent with the results of relevant basic studies (26, 27). Intra-abdominal hypertension will directly force the diaphragm to rise toward the thoracic cavity, decreasing the initial length of the diaphragm. According to the Frank-Starling law, muscle fibers contract at an unfavorable initial length, and the generated force will significantly reduce. At the same time, the continuous increase in intra-abdominal pressure will compress aortic and diaphragmatic microvessels, reducing the blood perfusion of the diaphragm, inducing local ischemia and hypoxia of the diaphragm, and aggravating diaphragm dysfunction (26, 27). In addition, mechanical ventilation is an independent risk factor for intra-abdominal hypertension, and the two conditions form a vicious circle, aggravating diaphragm dysfunction (28). Liu et al. (29) also found that the incidence of atelectasis in elderly patients undergoing general anesthesia with artificial pneumoperitoneum is closely associated with the degree of diaphragm inhibition. Therefore, medical staff should closely monitor the intra-abdominal pressure of mechanically ventilated patients, evaluate diaphragm function in combination with diaphragm ultrasound, take active intervention measures for high-risk groups with intra-abdominal hypertension, and treat the mechanical damage of the diaphragm in a timely manner once the intra-abdominal pressure is maintained at ≥16 mmHg.

#### 4.2.3 Patients with controlled mechanical ventilation are prone to early VIDD

This study indicated that within 24 h of mechanical ventilation, the risk of VIDD in patients with controlled mechanical ventilation was 11.45 times that of patients with assisted mechanical ventilation, which is consistent with the results of previous studies (30, 31). Mechanical ventilation is a major driver of VIDD. In particular, the damaging effect of controlled mechanical ventilation is greater. In this mode, the ventilator completely drives the forcible breathing of patients, and the diaphragm cannot afford active contraction, thereby causing disuse atrophy (32). At the same time, controlled ventilation is more likely to impair the synchrony of diaphragm contraction, resulting in abnormal excessive traction or resistant contraction, leading to diaphragmatic microdamage (32). The latest clinical trials have shown that continuous on-demand phrenic nerve stimulation-assisted mechanical ventilation can effectively prevent diaphragm disuse during mechanical ventilation (33). Therefore, medical staff should dynamically evaluate the functional status of the diaphragm and assess their respiratory drive capacity, preferentially selecting the assisted ventilation mode according to the actual situation of patients, optimizing respiratory treatment parameters, maintaining the appropriate respiratory drive of patients, and preventing diaphragm disuse. For critically ill patients without spontaneous breathing, neuroelectrical stimulation can be tried to induce diaphragm contraction and lower the risk of VIDD.

#### 4.2.4 Mechanically ventilated patients with complicated Sepsis are prone to early VIDD

This study revealed that the incidence of VIDD was significantly increased within the first 24 h of mechanical ventilation in patients with complicated sepsis. These findings are consistent with those of previous studies (34, 35). Sepsis and mechanical ventilation synergistically damage the structure and function of the diaphragm through several pathways within the first 24 h of mechanical ventilation. The systemic inflammatory response caused by sepsis directly damages diaphragmatic fibers and leads to calcium homeostasis imbalance and activation of proteolytic pathways, while mechanical ventilation leads to diaphragmatic disuse atrophy and microcirculation disturbance (35, 36). The combined effect of the two factors ultimately manifests as diaphragmatic fiber atrophy and significantly decreases contractility, leading to VIDD. Therefore, medical staff should identify whether mechanically ventilated patients are complicated with sepsis as early as possible, and administer effective antibiotics, anti-inflammatory agents, and other necessary drugs in a timely manner to minimize diaphragmatic damage caused by sepsis and improve the prognosis of patients.

#### 4.2.5 Mechanically ventilated patients with RASS ≤−4 are prone to early VIDD

The RASS score is a clinically standardized tool for evaluating sedation and agitation levels, and RASS ≤−4 suggests that the patient is in a deep sedation state (37). This study revealed that the risk of VIDD significantly increases within the first 24 h of deeply sedated mechanical ventilation, which was consistent with the results of Capdevila et al. (38). Sedative drugs can significantly inhibit the respiratory center of patients, leading to the weakness or even absence of diaphragmatic electrical activity, delaying the recovery of diaphragm function (39). Reduced respiratory effort caused by sedation will lead to diaphragmatic disuse atrophy, ultimately causing functional disorders (40). Respiratory depression and diaphragm dysfunction caused by deep sedation are more severe (41). Even if the patient’s sedation depth meets the standard, their respiratory drive may present various states, such as decreased, moderate, or excessive increase. In clinical practice, medical staff often administer empirical deep sedation to patients in the early phases of mechanical ventilation to reduce the spontaneous breathing efforts of patients (42). Therefore, medical staff should closely monitor the respiratory drive and inspiratory effort of mechanically ventilated patients, balance the relationship between respiratory drive and inspiratory effort, and adopt strategies such as sedative titration and daily interruption of sedation (41) to avoid empirical excessive sedation and reduce diaphragmatic disuse injury.

The aforementioned five risk factors do not exist alone. A high APACHE II score and sepsis constitute the patient’s susceptible background, intra-abdominal hypertension affects diaphragm function from the mechanical aspect, while controlled ventilation and deep sedation impair diaphragm activity from the functional aspect, which synergistically contribute to the development of early VIDD through multiple dimensions. Notably, while the RASS score (deep sedation) and controlled ventilation mode are clinically associated—deeply sedated patients often lack spontaneous respiratory drive and are consequently placed on controlled ventilation—collinearity diagnostics (VIF < 5) confirmed that each variable contributed independent predictive information. This statistical independence suggests that sedation strategy and ventilator mode selection represent separate clinical decision points that can both be targeted for intervention. It is also worth discussing the temporal relationship between the APACHE II score and the outcome. APACHE II is calculated using the worst values within the first 24 ICU hours, while VIDD is also diagnosed within 24 h of ventilation. This temporal overlap could theoretically introduce circularity. However, in practice, many APACHE II components are available within hours of ICU admission, often before significant diaphragm dysfunction manifests. The inclusion of APACHE II reflects the clinical reality that severe acute illness predisposes patients to rapid diaphragm injury, and future studies could further refine this by capturing even earlier timepoint data (e.g., within 2 h of ICU arrival).

### 4.3 Absence of ultrasound predictors and the model’s clinical rationale

A key finding requiring explicit discussion is that no diaphragm ultrasound-derived indicator was retained in the final nomogram. This does not diminish the role of ultrasound; rather, it reflects our deliberate study design. We employed diaphragm ultrasound as the gold standard to define the outcome (VIDD) with high specificity. The ultrasound indicators (TFdi, DE, Tdi) are themselves components of the diagnostic criteria. Including them as predictors of the outcome they define would constitute circular reasoning. This design choice means our model is intended to function as a pre-ultrasound screening tool. It enables bedside clinicians, and particularly nursing staff, to rapidly identify high-risk patients based on five instantly available clinical parameters. In settings where 24/7 expert ultrasound coverage is unavailable, or when a physician’s ultrasound order is pending, this nomogram provides an evidence-based prompt to initiate diaphragm-protective strategies without delay.

### 4.4 Model performance and clinical utility

The nomogram demonstrated good discriminative ability (AUC > 0.8) and calibration in both modeling and temporal validation cohorts. The five predictors—sepsis, controlled ventilation, RASS ≤ − 4, APACHE II > 20, and IAP ≥ 16 mmHg—are all rapidly obtainable within the first hours of ICU admission. For clinical implementation, we propose a two-step workflow: Step 1 (Hour 0–2): Upon initiating mechanical ventilation, the bedside nurse or resident calculates the nomogram score using data already available in the electronic health record. Step 2: For patients identified as high-risk (e.g., predicted probability > 0.48, the optimal cutoff), a “VIDD Prevention Bundle” is triggered. This bundle includes: (a) immediate discussion with the attending physician about switching from controlled to assist ventilation, if clinically feasible; (b) a pharmacist- and physician-involved sedation protocol review to target light sedation (RASS −2 to 0) or daily sedation interruption; (c) immediate IAP monitoring, with intervention if ≥16 mmHg; and (d) a “diaphragm-protective” alert placed on the nursing care plan to ensure vigilance during all patient handling procedures. We emphasize that this proposed workflow is hypothesis-generating and requires prospective clinical validation. The nomogram is intended for risk stratification, not as a standalone diagnostic tool, and all interventions should be guided by comprehensive clinical judgment.

### 4.5 Limitations

This study had several limitations that should be acknowledged. First, it was a single-center study conducted in a tertiary hospital in Shenzhen. Patient demographics, ICU case-mix, and clinical practices may differ from those in primary or secondary hospitals and in other geographic regions, which limits the generalizability of the model. Independent external validation in geographically and clinically diverse multicenter cohorts is required before wide clinical implementation. Second, the temporal validation cohort (*n =* 142) comprised a smaller sample than the modeling cohort. Although the AUC confidence interval (0.748–0.885) indicates acceptable precision for the validation AUC, a larger sample would provide more stable estimates and greater statistical power for sensitivity analyses. Third, the study only focused on the 24-h golden window of mechanical ventilation for early VIDD prediction and did not conduct a long-term follow-up study to explore the predictive value of the model for VIDD progression and long-term patient outcomes (e.g., weaning success rate, length of hospital stay, and long-term mortality). Fourth, the model only included clinical and ventilation-related indicators and did not integrate molecular biomarkers (e.g., muscle atrophy-related cytokines and oxidative stress indicators) that may be associated with VIDD. This may limit the predictive accuracy of the model. Fifth, as discussed above, while statistically independent, the clinical overlap between deep sedation and controlled ventilation mode should be recognized. Future multi-center, large-sample studies, incorporating molecular biomarkers and longitudinal outcomes, are needed to validate and refine this model and to test whether model-guided interventions can directly reduce VIDD incidence and improve patient prognosis.

## 5 Conclusion

In summary, complicated sepsis, controlled mechanical ventilation mode, deep sedation (RASS score ≤ − 4), an APACHE II score >20 within 24 h of admission, and complicated intra-abdominal hypertension [intra-abdominal pressure (IAP) ≥ 16 mmHg] are independent risk factors for VIDD within the first 24 h of mechanical ventilation. The nomogram constructed from these five readily available clinical variables demonstrated robust discriminative and calibration performance in both modeling and temporal validation cohorts. Importantly, this model is designed not to replace diaphragm ultrasound, but to complement it by enabling risk stratification at the earliest possible moment—before ultrasound examination is performed. This tool empowers nurses and physicians to promptly initiate diaphragm-protective interventions, including optimization of ventilation modes, sedation titration, and intra-abdominal pressure management, within the critical ultra-early window of mechanical ventilation.

## Data Availability

The raw data supporting the conclusions of this article will be made available by the authors, without undue reservation.
